# Antihypertensive Medication Class and Functional Outcomes After Nonlobar Intracerebral Hemorrhage

**DOI:** 10.1001/jamanetworkopen.2024.57770

**Published:** 2025-02-03

**Authors:** Mohamed Ridha, James F. Burke, Padmini Sekar, Daniel Woo, Yousef Hannawi

**Affiliations:** 1 Department of Neurology, The Ohio State University, Columbus; 2 Department of Neurology, University of Cincinnati, Cincinnati, Ohio

## Abstract

**Importance:**

Hypertension is the predominant pathology underlying nonlobar intracerebral hemorrhage (ICH), and antihypertensive agents have distinct biological implications for cerebral microvasculature. It is unknown if the class of antihypertensive medications initiated after ICH affects functional outcome beyond blood pressure (BP) control.

**Objective:**

To ascertain the association between the class of antihypertensive agents initiated during hospitalization and 90-day functional outcome in nonlobar ICH.

**Design, Setting, and Participants:**

This cohort study uses data from the Ethnic/Racial Variations of Intracerebral Hemorrhage (ERICH) study, a case-control cohort study investigating ICH risk factors among Hispanic, non-Hispanic Black (hereafter Black), and non-Hispanic White (hereafter White) populations at 42 US hospitals from 2010 to 2015. Data for this analysis were examined from May to September 2024. ERICH study participants were selected for the present analysis if they survived hospitalization and had available covariate and outcome data. Individuals with complications that would limit antihypertensive choice were excluded.

**Exposures:**

Initiation of angiotensin-converting enzyme inhibitor (ACEI) or angiotensin receptor blocker (ARB), calcium channel blocker, β-blocker, thiazide diuretic, and other antihypertensive medications during index hospitalization.

**Main Outcomes and Measures:**

Primary outcome was a favorable functional outcome, defined as a 90-day (follow-up) modified Rankin Score score of 0 to 2 (score range: 0 [indicating no disability] to 6 [indicating death]). Mixed-effects logistic regression adjusted for demographic characteristics, medical history, ICH characteristics, BP measurement, total number of antihypertensive medications, and hospitalization site was used to calculate the odds of favorable functional outcome.

**Results:**

Of the 1561 ERICH study participants in the analytic cohort, 1079 had nonlobar and 482 had lobar ICH. Among the 1079 participants in the nonlobar ICH group (mean [SD] age, 58.5 [12.9] years; 676 males [62.6%]; 429 Hispanic [39.8%], 388 Black [36.0%], and 262 White [24.4%] individuals), a total of 407 (37.7%) ACEIs or ARBs, 419 (38.8%) β-blockers, 503 (46.6%) calcium channel blockers, 180 (16.7%) thiazide diuretics, and 277 (25.7%) other antihypertensive classes were initiated during hospitalization (median [IQR], 3 [2-3] agents at discharge). At follow-up, 481 participants (44.6%) had a favorable functional outcome. Initiation of ACEI or ARB was associated with higher odds of favorable functional outcome (adjusted OR [AOR], 1.49; 95% CI, 1.08-2.05; *P* = .01). No other antihypertensive class was associated with functional outcome. Findings were consistent across several sensitivity analyses. The interaction with ACEI or ARB was mediated by the presence of radiographic features of cerebral small vessel disease (AOR, 3.04; 95% CI, 1.01-9.19; *P* = .049). No association with class of antihypertensive agent was observed in lobar ICH.

**Conclusions and Relevance:**

This large cohort study found that initiation of ACEI or ARB was associated with favorable 90-day functional outcomes after nonlobar ICH. This finding supports a medication class–specific benefit in hypertensive arteriopathy.

## Key Points

**Question:**

Is the class of oral antihypertensive agents initiated during hospitalization associated with the functional outcome in nonlobar spontaneous intracerebral hemorrhage?

**Findings:**

In this cohort study of 1079 patients with nonlobar intracerebral hemorrhage, the initiation of angiotensin-converting enzyme inhibitors or angiotensin II receptor blockers during hospitalization was associated with higher odds of favorable functional outcome. The magnitude of the association was greatest in patients with radiographic features of cerebral small vessel disease.

**Meaning:**

Findings of this study suggest a medication class–specific benefit in hypertensive arteriopathy; that is, antihypertensive agents targeting the renin-angiotensin system may be protective after hemorrhage attributed to hypertensive cerebral small vessel disease.

## Teaser

This cohort study examines the association between initiation of antihypertensive medications during hospitalization and functional outcomes at 90 days in patients with nonlobar intracerebral hemorrhage (ICH).

## Introduction

Intracerebral hemorrhage (ICH) accounts for approximately 15% of all strokes, with a high rate of long-term disability.^1^ Hypertension is the dominant modifiable risk factor, and long-term blood pressure (BP) control is effective in both primary and secondary prevention of ICH.^2,3^ Uncontrolled hypertension among survivors of ICH is associated with an increased risk of recurrent hemorrhage, cardiovascular events, and poor outcomes.^4,5^ Although acute BP control is routinely performed after ICH, optimal long-term antihypertensive regimens are unknown.^6^

Current guidelines recommend a long-term BP goal of 130/80 mm Hg after ICH; however, it is unknown whether antihypertensive class should be considered when initiating long-term agents.^7^ Medications inhibiting the renin-angiotensin system (RAS) may exhibit a BP-independent benefit in hypertensive cerebral small vessel disease (CSVD) by targeting underlying pathologic mechanisms, such as endothelial dysfunction and autoregulation impairment.^8,9^ Secondary analyses of randomized studies in high-risk hypertensive populations have suggested class-specific associations between RAS inhibitors and CSVD progression beyond BP control.^10,11^ Hypertensive CSVD accounts for the majority of nonlobar ICH, with a greater associated attributable risk of hypertension and burden of hypertension-associated alleles than lobar ICH.^2,12,13,14^ Therefore, we hypothesized that RAS-targeted agents would be associated with favorable functional outcomes after nonlobar ICH. Using data from the Ethnic/Racial Variations of Intracerebral Hemorrhage (ERICH) study, we sought to ascertain if the class of antihypertensive agents initiated during hospitalization was associated with 90-day functional outcome in nonlobar ICH.

## Methods

### Design and Population

The ERICH study was a multicenter, case-control cohort study that aimed to characterize racial and ethnic differences in ICH risk and characteristics among Hispanic, non-Hispanic Black (hereafter Black), and non-Hispanic White (hereafter White) populations at 42 US hospitals from 2010 to 2015. The complete study protocol has been previously published.^15^ The ERICH study received approval from the local institutional review board (IRB) at all sites. Written informed consent was obtained from all participants and/or designated proxies. This study was approved by The Ohio State University IRB. We followed the Strengthening the Reporting of Observational Studies in Epidemiology (STROBE) reporting guideline. Data for this analysis were examined from May to September 2024.

Details about the ERICH study population, data collection, and neuroimaging assessments are provided in the eMethods in Supplement 1. Participant selection for this analysis was restricted to individuals who survived beyond index hospitalization and had available covariate data and 90-day modified Rankin Scale (mRS) scores. Individuals with specific in-hospital complications that would limit antihypertensive medication choice were excluded. These complications included acute dehydration, acute heart failure exacerbation, pulmonary edema, and acute myocardial infarction. Enrolled participants who transitioned to comfort measures during the index hospitalization were excluded due to limitations on medical treatment initiation.

### Antihypertensive Class Initiation

Preadmission and discharge medication lists were abstracted from medical records. Medications were categorized according to current European and American guidelines on antihypertensive selection for the management of hypertension.^16,17^ Agents were classified as angiotensin-converting enzyme inhibitors (ACEIs), angiotensin receptor blockers (ARBs), calcium channel blockers (CCBs), β-blockers, thiazide diuretics, and other antihypertensive medications. Other antihypertensive agents included α2 agonists, α1 antagonists, nonthiazide diuretics, vasodilators, and nitrates. ACEIs and ARBs were categorized together, as simultaneous administration is contraindicated and both target the RAS. In-hospital initiation of an antihypertensive medication was defined as the presence of the medication class on discharge and absence on admission. Medication compliance was assessed at the 90-day follow-up interview.

### Outcomes

Participants were interviewed 90 days after ICH via telephone or in person to assess functional outcome using the mRS and Barthel Index for Activities of Daily Living.^18^ The mRS is a 7-point scale ranging from 0 (indicating no disability) to 6 (indicating death). The Barthel Index for Activities of Daily Living is a composite score of domains of activities of daily living, with 100 indicating full independence and 0 indicating complete dependence or death. The primary outcome was a favorable functional outcome, defined as a 90-day mRS score of 0 to 2.

### Statistical Analysis

Univariate associations of demographic and clinical characteristics with ICH location and functional outcome were evaluated using χ^2^ for categorical variables and Mann-Whitney test or unpaired, 2-tailed *t* test for continuous variables, as appropriate. Due to the inability to account for all confounding factors in selecting preadmission antihypertensive medications, hospital-initiated antihypertensive classes were chosen as the exposure variable for the primary analysis. This approach simulated a quasi-experimental design specific to hypertension management after ICH. Each antihypertensive class was input as unique binary variables.

A mixed-effects logistic regression model evaluated the association between antihypertensive class initiation and favorable functional outcome. Covariate selection for the model was made a priori, including factors associated with ICH outcomes and comorbidities affected by antihypertensive class: age, sex, race and ethnicity (self-reported in the ERICH study, which did not enroll people of American Indian or Alaska Native, Asian, Native Hawaiian or Other Pacific Islander, other, or unknown race and ethnicity), prior stroke, diabetes, coronary artery disease, atrial fibrillation, heart failure, creatinine level at admission, ICH volume (log-transformed), hematoma location (infratentorial vs deep), intraventricular hemorrhage (IVH) presence, mRS score prior to ICH, Glasgow Coma Scale (GCS; score range: 3 [indicating coma] to 15 [indicating normal responsiveness]) score at admission, average of 3 mean arterial pressures (MAPs) measured at enrollment interview, and total number of antihypertensive medications prescribed at discharge. Enrollment site was included as a random variable to account for individual institutional practice variation. Participants with missing covariate or outcome data were excluded from analysis. Planned sensitivity analyses are detailed in the eMethods in Supplement 1.

Post hoc analyses were planned to examine any antihypertensive class association identified from the primary analysis. For this purpose, logistic regression modeling using both theory-based and univariate statistics was used to derive individual-level estimated probabilities of antihypertensive class prescription at discharge. Inverse probability weighting was applied to all post hoc modeling. The first exploratory analysis tested the association of medication initiation, continuation, or discontinuation. The second exploratory analysis aimed to test the hypothesis that the benefit or harm of an antihypertensive class may be mediated by hypertensive CSVD. Both the main analysis and interaction between the initiated antihypertensive class and the presence of either cerebral microbleeds or moderate to severe white matter hyperintensities (WMHs) were assessed in a cohort of participants with available magnetic resonance imaging (MRI) data (radiographic measurement details are provided in the eMethods in Supplement 1). Additional post hoc analyses of the interaction between the number of antihypertensive classes initiated and race and ethnicity are described in the eMethods and eResults in Supplement 1. All post hoc analyses were adjusted for identical covariates of the primary model.

All reported *P* values were 2-sided, with the threshold of statistical significance set at *P* < .05. Statistical analysis was performed using SPSS, version 29 (IBM Corp).

## Results

### Participant Characteristics

Among 1561 included participants, 1079 had nonlobar ICH and 482 had lobar ICH (Table 1; eFigure 1 in Supplement 1). Excluded individuals had a greater prevalence of cardiac comorbidities, larger hematoma volume, and worse 90-day functional outcomes compared with participants (eTable 1 in Supplement 1). The analytic cohort (n = 1561) had a mean (SD) age of 60.1 (13.5) years and consisted of 645 females (41.3%) and 916 males (58.7%), of whom 505 identified as Black (32.4%), 594 as Hispanic (38.1%), and 462 as White (29.6%) individuals. Participants with nonlobar ICH (n = 1079) had a mean (SD) age of 58.5 (12.9) years and consisted of 403 females (37.3%) and 676 males (62.7%), of whom 388 identified as Black (36.0%), 429 as Hispanic (39.8%), and 262 as White (24.4%) individuals. Median (IQR) hematoma volume was 7.3 (2.9-15.1) mL, with IVH present in 444 individuals (41.1%) and infratentorial ICH in 186 (17.2%). Differences in demographics, comorbidities, and ICH characteristics between lobar and nonlobar ICH are shown in Table 1.

**Table 1.** Characteristics of Lobar and Nonlobar Intracerebral Hemorrhage

Characteristic	Participants, No. (%)		*P* value
	Nonlobar ICH (n = 1079)	Lobar ICH (n = 482)	
Age, mean (SD), y	58.5 (12.9)	63.7 (14.1)	<.001
Sex			
Female	403 (37.3)	242 (50.2)	<.001
Male	676 (62.6)	240 (49.8)	
Race and ethnicity^a^			
Black	388 (36.0)	117 (24.3)	
Hispanic	429 (39.8)	165 (34.2)	<.001
White	262 (24.3)	200 (41.5)	
Hypertension	895 (83.2)	363 (75.5)	<.001
Hyperlipidemia	300 (29.0)	179 (38.3)	<.001
Diabetes	301 (27.9)	139 (28.8)	.70
Atrial fibrillation	76 (7.0)	47 (9.8)	.07
Heart failure	74 (6.9)	33 (6.8)	.99
Coronary artery disease	113 (10.5)	79 (16.4)	.001
Prior stroke	158 (14.6)	93 (19.3)	.02
Statin use	234 (21.7)	145 (30.1)	<.001
Antiplatelet use	264 (24.5)	148 (30.7)	.01
Anticoagulant use	84 (7.8)	56 (11.6)	.01
Heavy alcohol use	169 (16.0)	47 (9.9)	.002
Active smoker	198 (18.4)	79 (16.5)	.35
Medical insurance	708 (66.5)	384 (80.0)	<.001
Transition to DNR status	30 (2.8)	26 (5.4)	.01
mRS score prior to ICH, median (IQR)	0 (0-0)	0 (0-1)	<.001
GCS score at admission, median (IQR)	15 (13-15)	15 (13-15)	.48
Hematoma volume, median (IQR), mL	7.3 (2.9-15.1)	18.4 (7.4-38.9)	<.001
IVH presence	444 (41.1)	124 (25.7)	<.001
Creatinine level at admission, median (IQR), mg/dL	1.0 (0.8-1.3)	0.9 (0.7-1.1)	<.001
In-hospital pneumonia	127 (11.8)	50 (1.4)	.42
Surgical evacuation	80 (7.4)	57 (11.8)	.005
MRI performed	464 (43.0)	263 (54.6)	<.001
Time to MRI, median (IQR), d	2 (1-3)	1 (1-3)	.27
Microbleeds presence	220 (52.6)^b^	116 (50.2)^c^	.56
Moderate to severe WMH presence	265 (57.7)^d^	137 (53.9)^e^	.33
BP measurement, mean (SD), mm Hg			
Admission systolic BP	190.2 (36.7)	175.4 (35.9)	<.001
Admission diastolic BP	106.5 (25.6)	94.7 (22.5)	<.001
Admission MAP	134.4 (27.2)	121.5 (24.8)	<.001
Enrollment systolic BP	137.7 (17.9)	135.9 (19.0)	.07
Enrollment diastolic BP	77.4 (12.4)	74.4 (12.9)	<.001
Enrollment MAP	97.5 (12.4)	94.9 (12.7)	<.001
Time to enrollment, median (IQR), d	7 (3-19)	7 (3-22)	.31
Time to follow-up, median (IQR), d	97 (84-110)	97 (86-110)	.80
Follow-up mRS score, median (IQR)	3 (2-4)	3 (1-4)	.06
Follow-up Barthel Index for Activities of Daily Living, median (IQR)^f^	85 (40-100)	90 (50-100)	.10
No. of antihypertensive medications prior to ICH, median (IQR)	0 (0-2)	1 (0-2)	.05
No. of antihypertensive medications at discharge, median (IQR)	3 (2-3)	2 (1-3)	<.001
No. of initiated antihypertensive medications, median (IQR)	2 (1-3)	1 (0-2)	<.001
Antihypertensive medications prior to ICH			
ACEI or ARB	329 (30.5)	179 (37.1)	.01
β-Blocker	266 (24.7)	136 (28.2)	.14
CCB	186 (17.2)	76 (15.8)	.47
Thiazide diuretic	126 (11.7)	55 (11.4)	.88
Other^g^	204 (18.9)	84 (17.4)	.49
Antihypertensive medications at discharge			
ACEI or ARB	676 (62.7)	258 (53.5)	<.001
β-Blocker	650 (60.2)	248 (51.5)	.001
CCB	670 (62.1)	194 (40.3)	<.001
Thiazide diuretic	249 (23.1)	76 (15.8)	.001
Other^g^	395 (36.6)	113 (23.4)	<.001
Antihypertensive medications initiated during hospitalization			
ACEI or ARB	407 (37.7)	120 (24.9)	<.001
β-Blocker	419 (38.8)	133 (27.6)	<.001
CCB	503 (46.6)	131 (27.2)	<.001
Thiazide diuretic	180 (16.7)	50 (10.4)	.001
Other^g^	277 (25.7)	61 (12.7)	<.001

Abbreviations: ACEI, angiotensin-converting enzyme inhibitor; ARB, angiotensin receptor blocker; BP, blood pressure; CCB, calcium channel blocker; DNR, do not resuscitate; GCS, Glasgow Coma Scale (score range: 3 [indicating coma] to 15 [indicating normal responsiveness]); ICH, intracerebral hemorrhage; IVH, intraventricular hemorrhage; MAP, mean arterial pressure; MRI, magnetic resonance imaging; mRS, modified Rankin Scale (score range: 0 [indicating no disability] to 6 [indicating death]); WMH, white matter hyperintensity.

SI conversion factor: To convert creatinine to micromoles per liter, multiply by 88.4.

^a^ Race and ethnicity were self-reported in the Ethnic/Racial Variations of Intracerebral Hemorrhage study. People identifying as American Indian or Alaska Native, Asian, Native Hawaiian or Other Pacific Islander, other, or unknown were not enrolled.

^b^ 418 Participants with data.

^c^ 231 Participants with data.

^d^ 459 Participants with data.

^e^ 137 Participants with data.

^f^ The Barthel Index for Activities of Daily Living is a composite score, with 100 indicating full independence and 0 indicating complete dependence or death.

^g^ Other antihypertensive agents included α2 agonists, α1 antagonists, nonthiazide diuretics, vasodilators, and nitrates.

Outcomes at 90 days were similar between nonlobar and lobar ICH. Participants with nonlobar ICH were less likely to receive an ACEI or ARB prior to admission and were discharged with more antihypertensive medications across all classes compared with participants with lobar ICH. Among participants with nonlobar ICH, the median (IQR) number of antihypertensive agents at discharge was 3 (2-3), with initiation of ACEI or ARB in 407 (37.7%), β-blockers in 419 (38.8%), CCBs in 503 (46.6%), thiazide diuretics in 180 (16.7%), and other agents in 277 (25.7%). Differences in antihypertensive class use between races and ethnicities are presented in eTable 2 in Supplement 1.

### Primary Outcome

A total of 481 participants (44.6%) with nonlobar ICH achieved an mRS score of 0 to 2 at follow-up. In univariate analysis, favorable functional outcomes were less common with older age, female sex, hyperlipidemia, statin use, diabetes, heart failure, prior stroke, insured status, transition to do not resuscitate status, higher premorbid mRS score, lower GCS score at admission, larger hematoma volume, IVH presence, neurosurgical intervention, higher initial systolic BP, moderate to severe WMHs, lower MAP at enrollment, and more antihypertensive medications on discharge. Favorable functional outcomes were more common in participants who initiated ACEI or ARB therapy (204 [42.4%] vs 203 [34.0%]; *P* = .004) and thiazide diuretic (95 [19.8%] vs 85 [14.2%]; *P* = .02) but were less common in participants who received β-blocker (164 [34.1%] vs 255 [42.6%]; *P* = .004), CCB (207 [43.0%] vs 296 [49.5%]; *P* = .03), or other medication classes (97 [20.8%] vs 180 [30.1%]; *P* < .001) (Table 2).

**Table 2.** Characteristics of Favorable or Unfavorable Outcome in Nonlobar Intracerebral Hemorrhage

Characteristic	Participants, No. (%)		*P* value
	Favorable outcome [mRS score: 0-2] (n = 481)	Unfavorable outcome [mRS score: 3-6] (n = 598)	
Age, mean (SD), y	55.9 (11.7)	60.5 (13.5)	<.001
Sex			
Female	161 (33.5)	242 (40.5)	.02
Male	320 (66.5)	356 (59.5)	
Race and ethnicity			
Black	179 (37.2)	209 (34.9)	.24
Hispanic	197 (41.0)	232 (38.8)	
White	105 (21.8)	157 (26.3)	
Hypertension	394 (82.1)	501 (84.1)	.39
Hyperlipidemia	119 (25.7)	181 (31.6)	.03
Diabetes	115 (23.9)	186 (31.1)	.009
Atrial fibrillation	28 (5.8)	48 (8.0)	.16
Heart failure	24 (5.0)	50 (8.4)	.03
Coronary artery disease	44 (9.1)	69 (11.5)	.20
Prior stroke	48 (10.0)	110 (18.4)	<.001
Statin use	91 (18.9)	143 (23.9)	.05
Antiplatelet use	109 (22.7)	155 (25.9)	.22
Anticoagulant use	36 (7.5)	48 (8.0)	.74
Heavy alcohol use	87 (18.3)	82 (14.1)	.06
Active smoker	84 (17.3)	115 (19.3)	.39
Medical insurance	290 (61.1)	418 (71.0)	<.001
Transition to DNR	3 (0.6)	27 (4.5)	<.001
mRS score prior to ICH, median (IQR)	0 (0-0)	0 (0-1)	<.001
GCS score at admission, median (IQR)	15 (14-15)	14 (10-15)	<.001
Hematoma volume, median (IQR), mL	4.4 (1.7-9.7)	10.3 (4.9-21.7)	<.001
ICH location			
Deep	401 (83.4)	492 (82.3)	.64
Infratentorial	80 (16.6)	106 (17.7)	
IVH presence	145 (30.2)	299 (50.0)	<.001
Creatinine level at admission, median (IQR), mg/dL	1.0 (0.8-1.3)	0.9 (0.8-1.3)	.35
In-hospital pneumonia	23 (4.8)	104 (17.4)	<.001
Surgical evacuation	11 (2.3)	69 (11.5)	<.001
MRI performed	236 (49.1)	228 (38.1)	<.001
Microbleeds presence	107 (48.4)^a^	113 (57.4)^b^	.07
Moderate to severe WMH presence	118 (50.2)^c^	147 (65.6)^d^	<.001
BP measurement, mean (SD), mm Hg			
Admission systolic BP	186.3 (37.2)	193.3 (36.1)	.003
Admission diastolic BP	106.1 (24.8)	106.8 (26.2)	.70
Admission MAP	132.9 (27.1)	135.6 (27.2)	.11
Enrollment systolic BP	137.7 (18.5)	137.6 (17.5)	.93
Enrollment diastolic BP	79.7 (12.5)	75.6 (11.9)	<.001
Enrollment MAP	99.0 (12.9)	96.2 (11.7)	<.001
No. of antihypertensive medications prior to ICH, median (IQR)	0 (0-2)	1 (0-2)	.10
No. of antihypertensive medications at discharge, median (IQR)	2 (2-3)	3 (2-4)	.02
No. of initiated antihypertensive medications, median (IQR)	2 (0-3)	2 (1-3)	<.001
Antihypertensive medications prior to ICH			
ACEI or ARB	136 (28.3)	193 (32.3)	.16
β-Blocker	110 (22.9)	156 (26.1)	.22
CCB	85 (17.7)	101 (16.9)	.74
Thiazide diuretic	59 (12.3)	67 (11.2)	.59
Other^e^	75 (15.6)	129 (21.6)	.01
Antihypertensive medications at discharge			
ACEI or ARB	323 (67.2)	353 (59.0)	.006
β-Blocker	260 (54.1)	390 (65.2)	<.001
CCB	287 (59.7)	383 (64.0)	.14
Thiazide diuretic	134 (27.9)	115 (19.2)	<.001
Other^e^	143 (29.7)	252 (42.1)	<.001
Antihypertensive medications initiated during hospitalization			
ACEI or ARB	204 (42.4)	203 (33.9)	.004
β-Blocker	164 (34.1)	255 (42.6)	.004
CCB	207 (43.0)	296 (49.5)	.03
Thiazide diuretic	95 (19.8)	85 (14.2)	.02
Other^e^	97 (20.2)	180 (30.1)	<.001

Abbreviations: ACEI, angiotensin-converting enzyme inhibitor; ARB, angiotensin receptor blocker; BP, blood pressure; CCB, calcium channel blocker; DNR, do not resuscitate; GCS, Glasgow Coma Scale (score range: 3 [indicating coma] to 15 [indicating normal responsiveness]); ICH, intracerebral hemorrhage; IVH, intraventricular hemorrhage; MAP, mean arterial pressure; MRI, magnetic resonance imaging; mRS, modified Rankin Scale (score range: 0 [indicating no disability] to 6 [indicating death]); WMH, white matter hyperintensity.

SI conversion factor: To convert creatinine to micromoles per liter, multiply by 88.4.

^a^ 221 Participants with data.

^b^ 197 Participants with data.

^c^ 235 Participants with data.

^d^ 224 Participants with data.

^e^ Other antihypertensive agents included α2 agonists, α1 antagonists, nonthiazide diuretics, vasodilators, and nitrates.

In the mixed-effects logistic regression model, age per year (adjusted odds ratio [AOR], 0.96; 95% CI, 0.94-0.97; *P* < .001), female sex (AOR, 0.73; 95% CI, 0.53-0.99; *P* = .04), prior stroke (AOR, 0.63; 95% CI, 0.39-0.99; *P* = .047), heart failure (AOR, 0.52; 95% CI, 0.27-1.00; *P* = .049), hematoma volume (AOR, 0.13; 95% CI, 0.09-0.20; *P* < .001), infratentorial ICH location (AOR, 0.67; 95% CI, 0.45-1.00; *P* = .049), IVH presence (AOR, 0.68; 95% CI, 0.50-0.93; *P* = .01), higher premorbid mRS score (AOR, 0.74; 95% CI, 0.62-0.89; *P* = .001), and lower GCS score at admission (AOR, 1.18; 95% CI, 1.11-1.25; *P* < .001) were associated with lower odds of favorable functional outcome. Initiation of ACEI or ARB was associated with greater odds of favorable functional outcomes (AOR, 1.49; 95% CI, 1.08-2.05; *P* = .01). Neither other antihypertensive medication class nor number of antihypertensive medications at discharge was associated with functional outcome (Table 3).

**Table 3.** Mixed-Effects Logistic Regression Model Analyzing Factors Associated With Favorable 90-Day Functional Outcome

Characteristic	AOR (95% CI)	*P* value
Age per y	0.96 (0.94-0.97)	<.001
Sex		
Female	0.73 (0.53-0.99)	.04
Male	1 [Reference]	NA
Race and ethnicity		
Black	0.96 (0.63-1.45)	.85
Hispanic	0.94 (0.63-1.40)	.77
White	1 [Reference]	NA
Prior stroke	0.63 (0.39-0.99)	.047
Diabetes	0.81 (0.57-1.14)	.22
Coronary artery disease	0.98 (0.58-1.64)	.94
Atrial fibrillation	1.50 (0.80-2.84)	.21
Heart failure	0.52 (0.27-1.00)	.049
Creatinine per mg/dL increase	1.03 (0.95-1.10)	.50
Log-transformed hematoma volume, baseline	0.13 (0.09-0.20)	<.001
ICH location (infratentorial vs deep)	0.67 (0.45-1.00)	.049
IVH presence	0.68 (0.50-0.93)	.01
mRS score prior to ICH	0.74 (0.62-0.89)	.001
GCS score at admission	1.18 (1.11-1.25)	<.001
MAP at enrollment per mm Hg increase	1.00 (0.99-1.02)	.58
Total No. of antihypertensive medications prescribed at discharge	0.93 (0.81-1.07)	.34
ACEI or ARB initiation	1.49 (1.08-2.05)	.01
β-Blocker initiation	0.82 (0.59-1.13)	.22
CCB initiation	0.80 (0.58-1.10)	.18
Thiazide diuretic initiation	1.01 (0.68-1.52)	.95
Other initiation^a^	0.79 (0.54-1.16)	.22

Abbreviations: ACEI, angiotensin-converting enzyme inhibitor; AOR, adjusted odds ratio; ARB, angiotensin receptor blocker; CCB, calcium channel blocker; GCS, Glasgow Coma Scale (score range: 3 [indicating coma] to 15 [indicating normal responsiveness]); ICH, intracerebral hemorrhage; IVH, intraventricular hemorrhage; MAP, mean arterial pressure; mRS, modified Rankin Scale (score range: 0 [indicating no disability] to 6 [indicating death]); NA, not applicable.

SI conversion factor: To convert creatinine to micromoles per liter, multiply by 88.4.

^a^ Other antihypertensive agents included α2 agonists, α2 antagonists, nonthiazide diuretics, vasodilators, and nitrates.

### Sensitivity Analyses

An alternative threshold of mRS score of 0 to 3 did not significantly modify the association of ACEI or ARB initiation with higher odds of favorable functional outcome (AOR, 1.49; 95% CI, 1.05-2.10; *P* = .02). Use of continuous mRS outcomes demonstrated the association of ACEI or ARB initiation with lower 90-day mRS score (β, −0.20; 95% CI, −0.37 to −0.03; *P* = .02). Among 1077 participants with an available 90-day Barthel Index for Activities of Daily Living, ACEI or ARB initiation was associated with greater functional independence (β, 0.20; 95% CI, 0.01-0.38; *P* = .04). No other antihypertensive medication was associated with alternative outcome measures (eTable 3 in Supplement 1). The unadjusted association of ACEI or ARB initiation with 90-day mRS score is illustrated in eFigure 2 in Supplement 1.

Sensitivity analyses excluding individuals who died prior to follow-up (n = 46), further adjusting for variables with univariate association, and specification of dihydropyridine CCB initiation did not change the association of ACEI or ARB initiation with higher odds of favorable functional outcome. Segregation of ACEI and ARB classes demonstrated that ARB initiation was associated with favorable functional outcomes (AOR, 2.66; 95% CI, 1.24-5.69; *P* = .01), while ACEI initiation did not meet the threshold of significance. Including all antihypertensive agents at discharge (newly initiated and continued medications), ACEI or ARB prescription was associated with higher odds of favorable function outcome (AOR, 1.84; 95% CI, 1.11-3.04; *P* = .02). Among 800 participants with medications at the 90-day follow-up, 247 (30.9%) were not compliant with initiated antihypertensive medications. For the remaining 553 participants (69.1%), ACEI or ARB initiation was associated with favorable functional outcome (AOR, 1.89; 95% CI, 1.17-3.08; *P* = .01), whereas CCB had lower odds of favorable functional outcome (AOR, 0.59; 95% CI, 0.37-0.96; *P* = .03). Among 1077 participants with available mRS scores on discharge, ACEI or ARB initiation was not associated with odds of favorable functional outcomes at discharge, whereas CCB (AOR, 0.67; 95% CI, 0.47-0.96; *P* = .03) or non–first-line antihypertensive (AOR, 0.49; 95% CI, 0.31-0.79; *P* = .003) initiation was associated with worse functional outcomes. Because no significant variation by site was observed in the primary analysis, a post hoc sensitivity analysis was performed using a traditional logistic regression model without adjustment for random effects, producing similar results (eTable 4 in Supplement 1).

### Lobar ICH

Of 482 participants with lobar ICH, 233 (48.3%) had a favorable functional outcome. Age per year (AOR, 0.97; 95% CI, 0.95-0.98; *P* < .001), prior stroke (AOR, 0.44; 95% CI, 0.23-0.83; *P* = .01), Hispanic ethnicity (AOR, 0.46; 95% CI, 0.27-0.78; *P* = .004), larger hematoma volume (AOR, 0.18; 95% CI, 0.11-0.32; *P* < .001), and higher premorbid mRS score (AOR, 0.60; 95% CI, 0.46-0.77; *P* < .001) were associated with lower odds of favorable functional outcomes. Neither the number nor class of antihypertensive medications was associated with functional outcomes (eTable 5 in Supplement 1).

### Continuation, Initiation, or Discontinuation of ACEI or ARB

The association between ACEI or ARB exposure and better functional outcome was explored in a prespecified post hoc analysis. Details of inverse probability weighting for ACEI or ARB prescription at discharge are provided in the eMethods in Supplement 1.

Of 1079 participants, 343 (31.8%) never received ACEI or ARB, 407 (37.7%) initiated ACEI or ARB, 269 (24.9%) continued ACEI or ARB, and 60 (5.6%) discontinued ACEI or ARB. Compared with participants with no exposure, ACEI or ARB initiation was associated with higher odds of favorable functional outcome (AOR, 2.14; 95% CI, 1.15-3.98; *P* = .02). Discontinuation was associated with lower odds of favorable functional outcomes compared with initiation (AOR, 0.21; 95% CI, 0.07-0.63; *P* = .005) and continuation (AOR, 0.25; 95% CI, 0.08-0.76; *P* = .02). No significant difference was observed between initiation and continuation of ACEI or ARB (Table 4). Adjusted estimated probabilities of no exposure, initiation, continuation, and discontinuation of ACEI or ARB are shown in the Figure.

**Table 4.** Post Hoc Exploratory Analyses of Association Between Renin-Angiotensin System Inhibitors and Outcomes

Exposure	AOR (95% CI)	*P* value
ACEI or ARB status^a^		
No exposure	1 [Reference]	NA
Initiation	2.14 (1.15-3.98)	.02
Continuation	1.80 (0.90-3.58)	.09
Discontinuation	0.44 (0.15-1.33)	.15
Interaction with CSVD^a^^,^^b^		
ACEI or ARB initiation	0.65 (0.25-1.70)	.38
CSVD feature present	0.45 (0.21-0.94)	.03
Interaction of ACEI or ARB initiation with CSVD	3.04 (1.01-9.19)	.049

Abbreviations: ACEI, angiotensin-converting enzyme inhibitor; AOR, adjusted odds ratio; ARB, angiotensin receptor blocker; CSVD, cerebral small vessel disease; NA, not applicable.

^a^ All results were from fully adjusted models.

^b^ CSVD features were defined as the presence of either moderate to severe white matter hyperintensity or microbleeds on brain magnetic resonance imaging.

**Figure.**
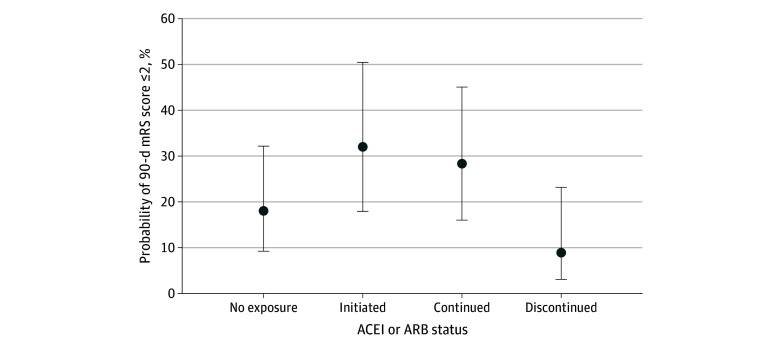
Estimated Probability of Favorable Functional Outcome by Angiotensin-Converting Enzyme Inhibitor (ACEI) or Angiotensin Receptor Blocker (ARB) Status Probabilities of achieving a modified Rankin Scale (mRS) score of 0 to 2 at 90 days were derived from fully adjusted regression model using inverse probability weighting. Error bars represent 95% CIs. mRS range: 0 (indicating no disability) to 6 (indicating death).

### Interaction Between CSVD

The interaction between ACEI or ARB initiation and CSVD was evaluated in 414 participants with characterized MRI features. Moderate to severe WMHs or cerebral microbleeds were detected in 294 participants. The presence of at least 1 CSVD feature was associated with lower odds of favorable functional outcome (AOR, 0.45; 95% CI, 0.21-0.94; *P* = .03). While an overall association of ACEI or ARB initiation with functional outcome was not observed, an interaction effect showed that ACEI or ARB initiation was associated with a higher odds of favorable functional outcome in the presence of CSVD (AOR, 3.04; 95% CI, 1.01-9.19; *P* = .049) (Table 4).

## Discussion

In this cohort study of the ERICH study data, we found that the initiation of RAS inhibitors was associated with favorable functional outcomes after nonlobar ICH, independent of BP control. No other antihypertensive class was associated with a 90-day functional outcome. This study found a potential class-specific association of RAS blockade with functional outcome after ICH attributed to hypertensive arteriopathy.

The biologic implications of angiotensin II for the cerebrovascular circulation has been extensively studied in animal models.^19,20^ Angiotensin II binding to the type 1 (AT1) receptor impairs endothelial function through induction of oxidative stress secondary to nicotinamide adenine dinucleotide phosphate oxidase activity.^21,22,23^ Targeted blockade of this pathway by ACEI or ARB improves endothelial-dependent cerebral autoregulation function.^24,25,26,27^ Although incompletely elucidated, endothelial dysfunction plays a role in the development of hypertensive CSVD.^8^ Conversely, angiotensin II binding at the type 2 (AT2) receptor promotes vasodilation and is potentially neuroprotective.^28,29^ In humans, analyses of several clinical trials have suggested that angiotensin II–inducing agents with selective blockade of AT1 provide superior cerebrovasculature protection and cognitive function.^30,31,32,33^ Although more participants in the ERICH study were initiated on ACEIs, the findings of this study support a potentially superior outcome with ARB initiation.

Given the role of angiotensin II in hypertension-induced cerebrovascular dysfunction, a plausible explanatory mechanism for the association between RAS-targeted agents and favorable functional outcomes after nonlobar ICH may be a salutary benefit for hypertensive CSVD. Diffusion-weighted imaging (DWI) lesions, potentially representing microinfarcts, are detected in approximately one-third of patients after ICH and are associated with poor outcomes.^34,35,36^ Although the mechanism is uncertain, prior investigations have suggested that DWI lesions may be an acute ischemic manifestation of hypertensive CSVD.^34^ A secondary analysis of both the PROGRESS (Perindopril Protection Against Recurrent Stroke Study) and the SPRINT (Systolic Blood Pressure Intervention Trial) demonstrated that RAS blockade prevents WMH progression to a greater extent than alternative antihypertensive medication regimens.^10,11^ As DWI lesions may occur beyond the acute period, prevention of microvascular ischemia may account for improved 90-day functional outcomes with ACEI or ARB initiation after nonlobar ICH.^37^ This hypothesis is supported by the association between functional outcome and ACEI or ARB and radiographic CSVD features in nonlobar ICH. It may also account for the absent association in lobar ICH, where hypertensive CSVD is not the predominant etiological cause.^13,14^ Because thrombotic events are common after ICH, it is also plausible that ACEI or ARB initiation may prevent other cardiovascular events, accounting for improved outcomes; however, we were not able to investigate this possibility.^38^

Several randomized clinical trials have investigated class-specific effects of RAS-targeted agents on stroke prevention. Both the HOPE (Heart Outcomes Prevention Evaluation) study and PROGRESS trial demonstrated superiority of ACEI-based regimens in stroke prevention among high-risk populations.^39,40^ The PROGRESS trial found that the combination of perindopril and indapamide reduced stroke recurrence, including ICH risk.^41,42^ The MOSES (Morbidity and Mortality After Stroke, Eprosartan Compared With Nitrendipine for Secondary Prevention) study demonstrated that ARB treatment in a hypertensive population who experienced a stroke was associated with reduced mortality and fewer cerebrovascular events compared with CCB.^43^ However, a BP-independent RAS blockade after ischemic stroke was not observed in the PROFESS (Prevention Regimen for Effectively Avoiding Second Strokes) trial.^44^ Subsequent meta-analyses have not reported an overall class-specific association of RAS inhibition with stroke prevention.^45,46,47^ Due to the heterogeneity of stroke mechanisms in these trials, further insight into downstream pathways of RAS may identify therapeutic targets specific to hypertensive CSVD beyond BP reduction.^9^

### Limitations

This study has several limitations. Due to the retrospective design, we were unable to exclude the possibility of residual confounders in medication selection or outcome. We adjusted for recruitment site to account for institution-specific treatment practices and performed multiple sensitivity analyses to assess identified potential confounders, with consistent results. Post hoc analysis demonstrated that ACEI or ARB continuation was concordant with initiation, whereas discontinuation was discordant, adding reassurance that our observation was not the result of type I statistical error. We did not observe an association between ACEI or ARB initiation and functional outcome at discharge to suggest that the 90-day functional outcome was attributable to bias from treatment patterns of inpatient clinicians. Other in-hospital factors, such as hyponatremia, arrythmia, acute kidney injury, and prior adverse medication effects, may have been associated with antihypertensive choice but data on these factors were not collected. It is possible that specific combinations of antihypertensive classes have synergistic properties. Although we did not observe an interaction between ACEI or ARB and the total number of agents, the retrospective nature of this study limited our insight into the benefits or harms of specific agents in combination with ACEI or ARB. Medication doses and adjustments may have affected results but were unavailable. Serial BP measurements were not collected over the follow-up period or at the 90-day period. Although ACEI or ARB had similar effectiveness in BP reduction compared with other first-line agents, the findings may be attributed to differential long-term BP control rather than to antihypertensive class.^16,17^ Because we excluded individuals with early withdrawal of care or with medical complications that limited antihypertensive selection, the cohort included those with less severe ICH and fewer cardiac comorbidities. Similarly, MRI acquisition for assessment of radiographic features of CSVD is frequently performed in participants with lower ICH severity.^48^ Additionally, functional outcome data were available for only 90 days in this study. It is recognized that neurologic recovery may be delayed after ICH, and an assessment at 6 months is likely a better reflection of long-term functional outcome.

## Conclusions

In this large cohort study of nonlobar ICH, ACEI or ARB initiation was associated with higher odds of favorable 90-day functional outcomes. This finding supports a medication class–specific benefit in hypertensive arteriopathy. Confirmatory and mechanistic studies are warranted to ascertain the therapeutic potential of RAS-targeted interventions after ICH.

## Notes

Supplement 1.
eMethods.

eResults

eReferences
**eTable 1.** Characteristics of Included and Excluded Participants **eTable 2.** Differences in Antihypertensive Classes Between Ethnic/Race Groups **eTable 3.** Alternative 90-Day Outcome Measures and Association With Antihypertensive Class **eTable 4.** Sensitivity Analyses of Association Between Antihypertensive Class With Favorable Outcome **eTable 5.** Association of Antihypertensive Class With Favorable Outcome in Lobar Intracerebral Hemorrhage **eFigure 1.** Flowchart of Cohort Derivation **eFigure 2.** Association Between Renin-Angiotensin System Inhibitor Initiation and Distribution of 90-Day Modified Rankin Scale After Non-Lobar Intracerebral Hemorrhage

Supplement 2.
Data Sharing Statement

## References

[1] Sheth KN. Spontaneous intracerebral hemorrhage. N Engl J Med. 2022;387(17):1589-1596. doi:10.1056/NEJMra2201449 36300975

[2] Martini SR, Flaherty ML, Brown WM, . Risk factors for intracerebral hemorrhage differ according to hemorrhage location. Neurology. 2012;79(23):2275-2282. doi:10.1212/WNL.0b013e318276896f 23175721 PMC3542348

[3] Walsh KB, Woo D, Sekar P, . Untreated hypertension: a powerful risk factor for lobar and nonlobar intracerebral hemorrhage in Whites, Blacks, and Hispanics. Circulation. 2016;134(19):1444-1452. doi:10.1161/CIRCULATIONAHA.116.024073 27737957 PMC5123682

[4] Biffi A, Teo KC, Castello JP, . Impact of uncontrolled hypertension at 3 months after intracerebral hemorrhage. J Am Heart Assoc. 2021;10(11):e020392. doi:10.1161/JAHA.120.020392 33998241 PMC8483505

[5] Biffi A, Anderson CD, Battey TWK, . Association between blood pressure control and risk of recurrent intracerebral hemorrhage. JAMA. 2015;314(9):904-912. doi:10.1001/jama.2015.10082 26325559 PMC4737594

[6] Mullen MT, Anderson CS. Review of long-term blood pressure control after intracerebral hemorrhage: challenges and opportunities. Stroke. 2022;53(7):2142-2151. doi:10.1161/STROKEAHA.121.036885 35657328

[7] Greenberg SM, Ziai WC, Cordonnier C, ; American Heart Association/American Stroke Association. 2022 Guideline for the management of patients with spontaneous intracerebral hemorrhage: a guideline from the American Heart Association/American Stroke Association. Stroke. 2022;53(7):e282-e361. doi:10.1161/STR.0000000000000407 35579034

[8] Santisteban MM, Iadecola C, Carnevale D. Hypertension, neurovascular dysfunction, and cognitive impairment. Hypertension. 2023;80(1):22-34. doi:10.1161/HYPERTENSIONAHA.122.18085 36129176 PMC9742151

[9] Iadecola C, Gorelick PB. Hypertension, angiotensin, and stroke: beyond blood pressure. Stroke. 2004;35(2):348-350. doi:10.1161/01.STR.0000115162.16321.AA 14757875

[10] Goldstein ED, Wolcott Z, Garg G, . Effect of antihypertensives by class on cerebral small vessel disease: a post hoc analysis of SPRINT-MIND. Stroke. 2022;53(8):2435-2440. doi:10.1161/STROKEAHA.121.037997 35506388

[11] Dufouil C, Chalmers J, Coskun O, ; PROGRESS MRI Substudy Investigators. Effects of blood pressure lowering on cerebral white matter hyperintensities in patients with stroke: the PROGRESS (Perindopril Protection Against Recurrent Stroke Study) Magnetic Resonance Imaging Substudy. Circulation. 2005;112(11):1644-1650. doi:10.1161/CIRCULATIONAHA.104.501163 16145004

[12] Kittner SJ, Sekar P, Comeau ME, . Ethnic and racial variation in intracerebral hemorrhage risk factors and risk factor burden. JAMA Netw Open. 2021;4(8):e2121921. doi:10.1001/jamanetworkopen.2021.21921 34424302 PMC8383133

[13] Georgakis MK, Gill D, Webb AJS, . Genetically determined blood pressure, antihypertensive drug classes, and risk of stroke subtypes. Neurology. 2020;95(4):e353-e361. doi:10.1212/WNL.0000000000009814 32611631 PMC7455321

[14] Meretoja A, Strbian D, Putaala J, . SMASH-U: a proposal for etiologic classification of intracerebral hemorrhage. Stroke. 2012;43(10):2592-2597. doi:10.1161/STROKEAHA.112.661603 22858729

[15] Woo D, Rosand J, Kidwell C, . The Ethnic/Racial Variations of Intracerebral Hemorrhage (ERICH) study protocol. Stroke. 2013;44(10):e120-e125. doi:10.1161/STROKEAHA.113.002332 24021679 PMC3873723

[16] Mancia G, Kreutz R, Brunström M, . 2023 ESH Guidelines for the management of arterial hypertension. The Task Force for the management of arterial hypertension of the European Society of Hypertension: endorsed by the International Society of Hypertension (ISH) and the European Renal Association (ERA). J Hypertens. 2023;41(12):1874-2071. doi:10.1097/HJH.0000000000003480 37345492

[17] Whelton PK, Carey RM, Aronow WS, . 2017 ACC/AHA/AAPA/ABC/ACPM/AGS/APhA/ASH/ASPC/NMA/PCNA Guideline for the prevention, detection, evaluation, and management of high blood pressure in adults: a report of the American College of Cardiology/American Heart Association Task Force on Clinical Practice Guidelines. Hypertension. 2018;71(6):e13-e115. doi:10.1161/HYP.0000000000000065 29133356

[18] Sulter G, Steen C, De Keyser J. Use of the Barthel Index and modified Rankin scale in acute stroke trials. Stroke. 1999;30(8):1538-1541. doi:10.1161/01.STR.30.8.1538 10436097

[19] Iadecola C, Gottesman RF. Neurovascular and cognitive dysfunction in hypertension. Circ Res. 2019;124(7):1025-1044. doi:10.1161/CIRCRESAHA.118.313260 30920929 PMC6527115

[20] Hannawi Y. Cerebral small vessel disease: a review of the pathophysiological mechanisms. Transl Stroke Res. 2024;15(6):1050-1069. doi:10.1007/s12975-023-01195-9 37864643

[21] Capone C, Faraco G, Park L, Cao X, Davisson RL, Iadecola C. The cerebrovascular dysfunction induced by slow pressor doses of angiotensin II precedes the development of hypertension. Am J Physiol Heart Circ Physiol. 2011;300(1):H397-H407. doi:10.1152/ajpheart.00679.2010 20971763 PMC3023263

[22] Girouard H, Park L, Anrather J, Zhou P, Iadecola C. Cerebrovascular nitrosative stress mediates neurovascular and endothelial dysfunction induced by angiotensin II. Arterioscler Thromb Vasc Biol. 2007;27(2):303-309. doi:10.1161/01.ATV.0000253885.41509.25 17138940

[23] Kazama K, Anrather J, Zhou P, . Angiotensin II impairs neurovascular coupling in neocortex through NADPH oxidase-derived radicals. Circ Res. 2004;95(10):1019-1026. doi:10.1161/01.RES.0000148637.85595.c5 15499027

[24] Davis LA, Smeda JS. Captopril treatment temporarily restores cerebral blood flow autoregulation in spontaneously hypertensive rats after hemorrhagic stroke. J Cardiovasc Pharmacol. 2010;56(3):255-262. doi:10.1097/FJC.0b013e3181e8af62 20531216

[25] Nishimura Y, Ito T, Saavedra JM. Angiotensin II AT_1_ blockade normalizes cerebrovascular autoregulation and reduces cerebral ischemia in spontaneously hypertensive rats. Stroke. 2000;31(10):2478-2486. doi:10.1161/01.STR.31.10.2478 11022082

[26] Schiffrin EL, Park JB, Intengan HD, Touyz RM. Correction of arterial structure and endothelial dysfunction in human essential hypertension by the angiotensin receptor antagonist losartan. Circulation. 2000;101(14):1653-1659. doi:10.1161/01.CIR.101.14.1653 10758046

[27] Ando H, Zhou J, Macova M, Imboden H, Saavedra JM. Angiotensin II AT_1_ receptor blockade reverses pathological hypertrophy and inflammation in brain microvessels of spontaneously hypertensive rats. Stroke. 2004;35(7):1726-1731. doi:10.1161/01.STR.0000129788.26346.18 15143297

[28] Fernandez LA, Caride VJ, Strömberg C, Näveri L, Wicke JD. Angiotensin AT_2_ receptor stimulation increases survival in gerbils with abrupt unilateral carotid ligation. J Cardiovasc Pharmacol. 1994;24(6):937-940. doi:10.1097/00005344-199424060-00011 7898077

[29] Li J, Culman J, Hörtnagl H, . Angiotensin AT2 receptor protects against cerebral ischemia-induced neuronal injury. FASEB J. 2005;19(6):617-619. doi:10.1096/fj.04-2960fje 15665034

[30] Hajjar I, Okafor M, McDaniel D, . Effects of candesartan vs lisinopril on neurocognitive function in older adults with executive mild cognitive impairment: a randomized clinical trial. JAMA Netw Open. 2020;3(8):e2012252. doi:10.1001/jamanetworkopen.2020.12252 32761160 PMC7411539

[31] Marcum ZA, Cohen JB, Zhang C, ; Systolic Blood Pressure Intervention Trial (SPRINT) Research Group. Association of antihypertensives that stimulate vs inhibit types 2 and 4 angiotensin II receptors with cognitive impairment. JAMA Netw Open. 2022;5(1):e2145319. doi:10.1001/jamanetworkopen.2021.45319 35089354 PMC8800076

[32] Fournier A, Messerli FH, Achard JM, Fernandez L. Cerebroprotection mediated by angiotensin II: a hypothesis supported by recent randomized clinical trials. J Am Coll Cardiol. 2004;43(8):1343-1347. doi:10.1016/j.jacc.2003.10.060 15093864

[33] Boutitie F, Oprisiu R, Achard JM, . Does a change in angiotensin II formation caused by antihypertensive drugs affect the risk of stroke? a meta-analysis of trials according to treatment with potentially different effects on angiotensin II. J Hypertens. 2007;25(8):1543-1553. doi:10.1097/HJH.0b013e32814a5ae5 17620946

[34] Murthy SB, Cho SM, Gupta A, . A pooled analysis of diffusion-weighted imaging lesions in patients with acute intracerebral hemorrhage. JAMA Neurol. 2020;77(11):1390-1397. doi:10.1001/jamaneurol.2020.2349 32687564 PMC7372494

[35] Ter Telgte A, Scherlek AA, Reijmer YD, . Histopathology of diffusion-weighted imaging-positive lesions in cerebral amyloid angiopathy. Acta Neuropathol. 2020;139(5):799-812. doi:10.1007/s00401-020-02140-y 32108259 PMC7185568

[36] Kidwell CS, Rosand J, Norato G, . Ischemic lesions, blood pressure dysregulation, and poor outcomes in intracerebral hemorrhage. Neurology. 2017;88(8):782-788. doi:10.1212/WNL.0000000000003630 28122903 PMC5344081

[37] Menon RS, Burgess RE, Wing JJ, . Predictors of highly prevalent brain ischemia in intracerebral hemorrhage. Ann Neurol. 2012;71(2):199-205. doi:10.1002/ana.22668 22367992 PMC3298034

[38] Li L, Murthy SB. Cardiovascular events after intracerebral hemorrhage. Stroke. 2022;53(7):2131-2141. doi:10.1161/STROKEAHA.122.036884 35674043 PMC9247019

[39] Heart Outcomes Prevention Evaluation Study Investigators. Effects of ramipril on cardiovascular and microvascular outcomes in people with diabetes mellitus: results of the HOPE study and MICRO-HOPE substudy. Lancet. 2000;355(9200):253-259. doi:10.1016/S0140-6736(99)12323-7 10675071

[40] PROGRESS Collaborative Group. Randomised trial of a perindopril-based blood-pressure-lowering regimen among 6,105 individuals with previous stroke or transient ischaemic attack. Lancet. 2001;358(9287):1033-1041. doi:10.1016/S0140-6736(01)06178-5 11589932

[41] Chapman N, Huxley R, Anderson C, ; Writing Committee for the PROGRESS Collaborative Group. Effects of a perindopril-based blood pressure–lowering regimen on the risk of recurrent stroke according to stroke subtype and medical history: the PROGRESS trial. Stroke. 2004;35(1):116-121. doi:10.1161/01.STR.0000106480.76217.6F 14671247

[42] Arima H, Tzourio C, Anderson C, ; PROGRESS Collaborative Group. Effects of perindopril-based lowering of blood pressure on intracerebral hemorrhage related to amyloid angiopathy: the PROGRESS trial. Stroke. 2010;41(2):394-396. doi:10.1161/STROKEAHA.109.563932 20044530

[43] Schrader J, Lüders S, Kulschewski A, ; MOSES Study Group. Morbidity and Mortality After Stroke, Eprosartan Compared With Nitrendipine for Secondary Prevention: principal results of a prospective randomized controlled study (MOSES). Stroke. 2005;36(6):1218-1224. doi:10.1161/01.STR.0000166048.35740.a9 15879332

[44] Yusuf S, Diener HC, Sacco RL, ; PRoFESS Study Group. Telmisartan to prevent recurrent stroke and cardiovascular events. N Engl J Med. 2008;359(12):1225-1237. doi:10.1056/NEJMoa0804593 18753639 PMC2714258

[45] Wei J, Galaviz KI, Kowalski AJ, . Comparison of cardiovascular events among users of different classes of antihypertension medications: a systematic review and network meta-analysis. JAMA Netw Open. 2020;3(2):e1921618. doi:10.1001/jamanetworkopen.2019.21618 32083689 PMC7043193

[46] Wang WT, You LK, Chiang CE, . Comparative effectiveness of blood pressure-lowering drugs in patients who have already suffered from stroke: traditional and bayesian network meta-analysis of randomized trials. Medicine (Baltimore). 2016;95(15):e3302. doi:10.1097/MD.0000000000003302 27082571 PMC4839815

[47] Rashid P, Leonardi-Bee J, Bath P. Blood pressure reduction and secondary prevention of stroke and other vascular events: a systematic review. Stroke. 2003;34(11):2741-2748. doi:10.1161/01.STR.0000092488.40085.15 14576382

[48] Ridha M, Hannawi Y, Murthy S, . Premorbid blood pressure control modifies risk of DWI lesions with acute blood pressure reduction in intracerebral hemorrhage. Hypertension. 2024;81(10):2113-2123. doi:10.1161/HYPERTENSIONAHA.124.23271 39069917 PMC11410531

