# Supplementary material for: Antihypertensive Medication Class and Functional Outcomes After Nonlobar Intracerebral Hemorrhage
Source: JAMA Netw Open. 2025 Feb 3;8(2):e2457770. doi: 10.1001/jamanetworkopen.2024.57770 (PMC11791703; doi:10.1001/jamanetworkopen.2024.57770)
Supplement: Supplement 1. — eMethods. eResults eReferences eTable 1. Characteristics of Included and Excluded Participants eTable 2. Differences in Antihypertensive Classes Between Ethnic/Race Groups eTable 3. Alternative 90-Day Outcome Measures and Association With Antihypertensive Class eTable 4. Sensitivity Analyses of Association Between Antihypertensive Class With Favorable Outcome eTable 5. Association of Antihypertensive Class With Favorable Outcome in Lobar Intracerebral Hemorrhage eFigure 1. Flowchart of Cohort Derivation eFigure 2. Association Between Renin-Angiotensin System Inhibitor Initiation and Distribution of 90-Day Modified Rankin Scale After Non-Lobar Intracerebral Hemorrhage [file jamanetwopen-e2457770-s001.pdf]

Supplemental Online Content

Ridha M, Burke JF, Sekar P, Woo D, Hannawi Y. Antihypertensive medication class and functional outcomes after nonlobar intracerebral hemorrhage. *JAMA Netw Open*. 2025;8(2):e2457770. doi:10.1001/jamanetworkopen.2024.57770

eMethods

eResults

eReferences

eTable 1. Characteristics of Included and Excluded Participants

eTable 2. Differences in Antihypertensive Classes Between Ethnic/Race Groups

eTable 3. Alternative 90-Day Outcome Measures and Association With Antihypertensive Class

eTable 4. Sensitivity Analyses of Association Between Antihypertensive Class With Favorable Outcome

eTable 5. Association of Antihypertensive Class With Favorable Outcome in Lobar Intracerebral Hemorrhage

eFigure 1. Flowchart of Cohort Derivation

eFigure 2. Association Between Renin-Angiotensin System Inhibitor Initiation and Distribution of 90-Day Modified Rankin Scale After Non-Lobar Intracerebral Hemorrhage

This supplemental material has been provided by the authors to give readers additional information about their work.

© 2025 Ridha M et al. *JAMA Network Open.*

eMethods

ERICH Study Population

The primary objective of the ERICH study was to understand the disproportionate ICH risk among non-Hispanic blacks and Hispanics compared to non-Hispanic whites in the United States. Inclusion criteria for the study required participants be from one of three self-reported race/ethnic groups: Hispanic, non-Hispanic black, and non-Hispanic white. Each participant was designated to one of these categories within the original dataset. Asians, non-Hispanic indigenous groups, Pacific Islanders, unknown race/ethnic groups, or other were not enrolled. Although not evaluated in this current analysis, national origin was collected in the ERICH study for Hispanics and non-Hispanic blacks. Among non-Hispanic black, national origin categories included African, African American, African European, Bahamian, Haitian, Jamaican, Trinidadian-Tobagonian, unknown/unspecified, or other. Among Hispanics, national origin was recorded as Dominican, Central American, Cuban, Mexican, Puerto Rican, South American, unknown, or other. The ERICH study enrolled a total of 1000 non-Hispanic white, non-Hispanic black, and Hispanic adults with spontaneous ICH. An equal number of demographically matched ICH-free adults were recruited as controls. Cases were defined by the presence of intraparenchymal blood on initial brain imaging in individuals presenting with acute neurologic deficits. Eligible participants were identified via “hot pursuit” with active screening of institutional admission records to minimize survival bias. ICH due to secondary causes such as trauma, brain tumors, venous sinus thrombosis, ischemic stroke, or vascular malformations were excluded. The study was observational, and treatment practices were provider specific. The eligibility criteria is detailed in the study protocol.[1]

© 2025 Ridha M et al. *JAMA Network Open.*

Data Collection

Demographics, medical history, cardiovascular risk factors, initial BP measurement, initial Glasgow Coma Scale (GCS), laboratory results, surgical hematoma evacuation, and in-hospital complications were abstracted from hospitalization records. Ethnic/race group was assessed to account for known differences in ICH characteristics, risk factors, and outcomes. Pre-morbid and discharge mRS were abstracted for each subject. In-hospital events including change in code status, pneumonia, heart failure exacerbation or pulmonary edema, dehydration, acute myocardial infarction were determined by a documented diagnosis from the clinical team. Active cigarette smoking, moderate-heavy alcohol use (defined as greater than two drinks daily), and medical insurance were obtained from in-person study interview. All subjects and/or proxies underwent a standardized interview at enrollment which included three consecutive manual BP measurements using an appropriately sized cuff.

Neuroimaging Characteristics

All neuroimaging was adjudicated by the central imaging core. Hematoma volume, the presence of intraventricular hemorrhage (IVH), and hematoma location determined from the initial non-contrast computerized tomography (CT) image. Intraparenchymal hematoma volume was calculated using planimetric analysis. Hematoma location was categorized into lobar, deep, and infratentorial hemorrhage. Magnetic resonance imaging (MRI) of the brain was obtained on 1.5-3.0 T scanners. Due to variations in local practices in MRI acquisition, the ERICH study mandated a research MRI to be obtained for every fifth subject if not performed clinically to minimize selection bias. Central adjudication of all MRIs was performed by a blinded study-

© 2025 Ridha M et al. *JAMA Network Open.*

adjudicator with expertise in cerebrovascular imaging using the following sequences: Fluid Attenuated Inversion Recovery (FLAIR), diffusion weighted imaging (DWI), apparent diffusion coefficient (ADC), and gradient echo (GRE). White matter hyperintensity (WMH) severity was graded on FLAIR imaging using a modified Fazekas score with moderate-severe WMH correlating with a Fazekas score of 2-3.[2] Cerebral microbleeds were identified on GRE imaging. For this analysis, the radiographic cerebral small vessel disease was identified as the presence of either cerebral microbleeds (included all locations and any numbers of lesions) or moderate-severe WMH.

Sensitivity Analyses

A series of sensitivity analyses were performed using alternative functional outcome measures: 1) favorable outcome redefined as mRS 0-3; 2)mRS as a continuous measure; 3) BI as a continuous measure. Additional sensitivity analyses were planned to address pre-specified potential confounders to minimize type-one error: 1) exclude subjects deceased at 90-day follow-up; 2) additional adjustment for covariates with significant univariate association with outcome; 3) restrict CCB initiation to dihydropyridine calcium channel blockers (DHP CCB); 4) segregate effects of ACEI and ARB initiation; 5) assess the associations all antihypertensives prescribed at discharge (continued and initiated medications); 6) exclude subjects not maintained on initiated antihypertensives at 90-day follow-up; 7) assess antihypertensive class association on discharge outcome. Mixed-effects linear regression models were utilized for analyses with continuous outcome measures. Due to the non-normal distribution of observed Barthel Index values, a gamma distribution was applied to the reflected dataset to permit model fitting. A secondary analysis was performed to assess the association of antihypertensive class initiation in lobar ICH.

© 2025 Ridha M et al. *JAMA Network Open.*

Post-hoc Analysis Inverse Probability Weighting

To account for confounders associated with the decision to prescribe ACEI or ARB on discharge, a logistic regression model was utilized to predict individual subject probability of being discharged on an ACEI or ARB. Variables with univariate associations with ACEI or ARB prescription at discharge included: age, sex, initial systolic BP (SBP), initial GCS, number of pre-admission antihypertensive agent, history of hypertension, prior stroke, diabetes hyperlipidemia, coronary artery disease, pre-admission use of ACEI or ARB, antiplatelet use, and statin use. The predictive model incorporated these variables in addition to non-acute MAP, atrial fibrillation, congestive heart failure, creatinine, ICH characteristics (hematoma volume, location, IVH), all pre-admission antihypertensive classes, transition to DNR, medical insurance status, tobacco use, alcohol use, functional outcome at discharge, and hospitalization site (random effect). Inverse probability weighting was applied in a mixed-effects logistic regression model to examine the association between initiation, continuation, discontinuation, or non-exposure of ACEI or ARB on outcomes, adjusting for identical covariates as the primary analysis.

Post-hoc Analyses Assessing Interaction Between ACEI or ARB Initiation With Additional Antihypertensive Classes and Ethnicity/Race

Two additional post-hoc analyses were performed to explore the potential interaction of combination regiments and ethnicity/race upon the association between ACEI or ARB use and favorable 90-day outcome. The former analysis input an interaction term between ACEI or ARB initiation and the total number of initiated classes in addition to the main effects. Due to the significant imbalances in pre-admission antihypertensive classes between race/ethnic groups, the

© 2025 Ridha M et al. *JAMA Network Open.*

latter analysis utilized an interaction term between ACEI or ARB prescribed at discharge and race/ethnic group.

© 2025 Ridha M et al. *JAMA Network Open.*

eResults

Interaction With Additional Antihypertensive Classes

Assessment of the interaction between ACEI or ARB initiation and the number of various initiated antihypertensive classes was performed to assess the potential impact of combination regimens. Of 1079 subjects with nonlobar ICH, 63 subjects were initiated on solely an ACEI or ARB while 344 received an ACEI or ARB with additional agents. Fully adjusted model results demonstrated a significant association between ACEI or ARB initiation with favorable outcomes (OR 3.31, CI 1.04-10.53, p=0.04). No significant interaction was detected between ACEI or ARB initiation and the total number of antihypertensive classes initiated (p=0.59).

Interaction With Ethnicity/Race

Exploration of the interaction between ACEI or ARB prescription at discharge and ethnic/race groups found no significant interaction effect between ACEI/ARB and ethnicity/race (p=0.901). Prescription of an ACEI or ARB at discharge was associated with favorable outcome (OR 2.94, CI 1.06-8.21, p=0.04).

eReferences

1. Woo D, Rosand J, Kidwell C, et al. The Ethnic/Racial Variations of Intracerebral Hemorrhage (ERICH) Study Protocol. *Stroke*. 2013;44(10):e120-e125. doi:10.1161/STROKEAHA.113.002332

2. Fazekas F, Barkhof F, Wahlund LO, et al. CT and MRI rating of white matter lesions. *Cerebrovasc Dis Basel Switz*. 2002;13 Suppl 2:31-36. doi:10.1159/000049147

© 2025 Ridha M et al. *JAMA Network Open.*

eTable 1. Characteristics of Included and Excluded Participants

	Participants, No. (%)		
**Characteristic**	**Included (n=1561)**	**Excluded (n=1439)**	**P-value**
Age, mean (SD)	60.1 (13.5)	63.6 (14.4)	<0.001
Female	645 (41.3)	590 (41.0)	0.86
Male	916 (58.7)	849 (59.0)	
Race and ethnicity			<0.001
Hispanic	594 (38.1)	406 (28.2)	
Non-Hispanic Black	505 (32.4)	495 (34.4)	
Non-Hispanic White	462 (29.6)	538 (37.4)	
Hypertension	1258 (80.8)	1149 (82.3)	0.31
Hyperlipidemia	479 (31.9)	445 (34.4)	0.16
Diabetes	440 (28.2)	390 (28.2)	0.97
Atrial fibrillation	123 (7.9)	166 (12.4)	<0.001
Heart failure	107 (6.9)	120 (9.0)	0.03
Coronary artery disease	192 (12.3)	209 (15.8)	0.007
Prior stroke	251 (16.1)	258 (18.4)	0.09
Statin use	379 (24.3)	353 (24.5)	0.87
Antiplatelet use	412 (26.4)	429 (29.8)	0.04
Anticoagulant use	140 (9.0)	177 (12.3)	0.003
Heavy alcohol	216 (14.1)	197 (14.1)	0.98
Active smoker	277 (17.8)	274 (19.3)	0.31
Medical insurance	1092 (70.7)	1104 (77.9)	<0.001
Transition to DNR	56 (3.6)	383 (26.6)	<0.001
Withdrawal of care	0 (0.0)	318 (22.1)	<0.001
In-hospital death	0 (0.0)	334 (23.3)	<0.001
mRS score prior to ICH, median (IQR)	0 (0-0)	0 (0-1)	<0.001
GCS score at admission, median (IQR)	15 (13-15)	14 (9-15)	<0.001
Hematoma volume, median (IQR), mL	9.4 (3.7-21.8)	13.3 (4.3-34.6)	<0.001
IVH presence	568 (36.4)	664 (49.4)	<0.001
ICH location			0.02
Lobar	482 (30.9)	430 (32.4)	
Deep	893 (57.2)	698 (52.6)	
Infratentorial	186 (11.9)	198 (14.9)	
Creatinine level at admission, median (IQR), mg/dL	0.9 (0.8-1.2)	1.00 (0.8-1.3)	0.003
In-hospital pneumonia	177 (11.3)	187 (13.0)	0.17
Surgical evacuation	137 (8.8)	129 (9.0)	0.86
MRI performed	727 (46.6)	473 (32.9)	<0.001
Microbleeds presence	336 (51.8)	336 (51.8)	0.40
Moderate to severe WMH presence	402 (56.4)	295 (63.9)	0.01
BP measurement, mean (SD), mm Hg			
Admission systolic BP	185.70 (37.1)	186.21 (38.5)	0.73
Admission diastolic BP	102.92 (25.3)	102.17 (25.9)	0.45
Admission MAP	130.49 (27.1)	130.17 (28.0)	0.76
Enrollment systolic BP	137.10 (18.3)	138.13 (29.9)	0.33
Enrollment diastolic BP	76.49 (12.5)	74.61 (27.6)	0.04
Enrollment MAP	96.69 (12.5)	95.79 (27.2)	0.32
Time to enrollment, median (IQR), d	7 (3-19)	5 (3-15)	<0.001
Time to follow-up, median (IQR), d	97 (85-110)	95 (81-109)	0.09
Follow-up mRS score, median (IQR)	3 (1-4)	4 (2-6)	<0.001
Follow-up Barthel Index, median (IQR)	90 (45-100)	40 (0-95)	<0.001

© 2025 Ridha M et al. *JAMA Network Open.*

	Patients, No. (%)		
Characteristic	Included (n=1561)	Excluded (n=1439)	P-value
No. of antihypertensive medications prior to ICH, median (IQR)	1 (0-2)	1 (0-2)	0.04
No. of antihypertensive medications at discharge, median (IQR)	2 (1-3)	2 (1-3)	0.67
Antihypertensive medications prior to ICH			
ACEI or ARB	508 (32.5)	448 (31.1)	0.41
β-blocker	402 (25.8)	424 (29.5)	0.02
CCB	262 (16.8)	263 (18.3)	0.28
Thiazide diuretic	181 (11.6)	165 (11.5)	0.91
Other	288 (18.5)	325 (22.6)	0.005
Antihypertensives at discharge			
ACEI or ARB	934 (59.8)	590 (58.5)	0.51
β-blocker	898 (57.5)	518 (51.0)	0.03
CCB	864 (55.4)	630 (61.8)	0.03
Thiazide diuretic	325 (20.8)	210 (14.6)	<0.001
Other	508 (32.5)	368 (36.5)	0.04
Antihypertensives initiated during hospitalization			
ACEI or ARB	527 (33.8)	345 (24.0)	<0.001
β-blocker	552 (35.4)	387 (26.9)	<0.001
CCB	634 (40.6)	365 (25.4)	<0.001
Thiazide diuretic	230 (14.7)	149 (10.4)	<0.001
Other	338 (21.7)	237 (23.5)	0.27

Abbreviations: ACEI, angiotensin converting enzyme inhibitor; ARB, angiotensin II receptor block; BP, blood pressure; CCB, calcium channel blocker; DNR, Do-Not-Resuscitate; GCS, Glasgow Coma Scale; ICH, intracerebral hemorrhage; IVH, intraventricular hemorrhage; MAP, mean arterial pressure; MRI, magnetic resonance imaging; mRS, modified Rankin Scale; WMH, white matter hyperintensity.

© 2025 Ridha M et al. *JAMA Network Open.*

eTable 2. Differences in Antihypertensive Classes Between Ethnic/Race Groups

	**Patients, No. (%)**			
**Characteristic**	**Hispanic (n=594)**	**Non-Hispanic Black (n=505)**	**Non-Hispanic White (n=462)**	**P-value**
Number of antihypertensives prior to ICH, median (IQR)	0 (0-1)	1 (0-2)	1 (0-2)	<0.001
Number of antihypertensives at discharge, median (IQR)	2 (1-3)	3 (2-4)	2 (1-3)	<0.001
Antihypertensives prior to ICH				
ACEI or ARB	173 (29.1)	157 (31.1)	178 (38.5)	0.004
β-blocker	120 (20.2)	141 (27.9)	141 (30.5)	<0.001
CCB	66 (11.1)	117 (23.2)	79 (17.1)	<0.001
Thiazide diuretic	48 (8.1)	82 (16.2)	51 (11.0)	<0.001
Other	88 (14.8)	102 (20.2)	98 (21.2)	0.014
Antihypertensives at discharge				
ACEI or ARB	366 (61.6)	292 (57.8)	276 (59.7)	0.44
β-blocker	320 (53.9)	327 (64.8)	251 (54.3)	<0.001
CCB	318 (53.5)	350 (69.3)	196 (42.4)	<0.001
Thiazide diuretic	113 (19.0)	146 (28.9)	66 (14.3)	<0.001
Other	180 (30.3)	199 (39.4)	129 (27.9)	<0.001
Antihypertensives initiated during hospitalization				
ACEI or ARB	232 (39.1)	175 (34.7)	120 (26.0)	<0.001
β-blocker	228 (38.4)	204 (40.4)	120 (26.0)	<0.001
CCB	258 (43.4)	247 (48.9)	129 (27.9)	<0.001
Thiazide diuretic	89 (15.0)	100 (19.8)	41 (8.9)	<0.001
Other	135 (22.7)	138 (27.3)	65 (14.1)	<0.001

Abbreviations: ACEI, angiotensin converting enzyme inhibitor; ARB, angiotensin II receptor block; CCB, calcium channel blocker; ICH, intracerebral hemorrhage

© 2025 Ridha M et al. *JAMA Network Open.*

eTable 3. Alternative 90-Day Outcome Measures and Association With Antihypertensive Class

**Association with mRS 0-3**		
**Exposure**	**AOR (95% CI)**	**P-value**
ACEI or ARB initiation	1.49 (1.05-2.10)	0.02
β-blocker initiation	0.85 (0.61-1.19)	0.34
CCB initiation	0.89 (0.64-1.26)	0.52
Thiazide diuretic initiation	1.31 (0.83-2.07)	0.25
Other class initiation	0.89 (0.60- 1.32)	0.57
**Association with mRS as a continuous measure**		
**Exposure**	**Adjusted Coefficient (95% CI)**	**P-value**
ACEI or ARB initiation	-0.20 (-0.371- -0.033)	0.02
β-blocker initiation	0.10 (-0.07- 0.27)	0.26
CCB initiation	0.13 (-0.04- 0.30)	0.12
Thiazide diuretic initiation	-0.16 (-0.37- 0.06)	0.15
Other class initiation	0.17 (-0.03- 0.37)	0.09
**Association with Barthel Index as a continuous measure**		
**Exposure**	**Adjusted Coefficient (95% CI)**	**P-value**
ACEI or ARB initiation	-0.20 (-0.39- -0.01)	0.04
β-blocker initiation	0.18 (-0.01- 0.37)	0.07
CCB initiation	0.08 (-0.11- 0.28)	0.39
Thiazide diuretic initiation	-0.20 (-0.44- 0.05)	0.12
Other class initiation	0.10 (-0.13- 0.33)	0.40

Abbreviations: ACEI, angiotensin converting enzyme inhibitor; ARB, angiotensin II receptor block; CCB, calcium channel blocker; mRS, modified Rankin Scale.

[a] Results of fully adjusted multivariable models are shown.

© 2025 Ridha M et al. *JAMA Network Open.*

eTable 4. Sensitivity Analyses of Association Between Antihypertensive Class With Favorable Outcome

Exposure	AOR (95% CI)	P-value
**Exclusion of deceased prior to 90-day follow up**		
ACEI or ARB initiation	1.47 (1.06- 2.03)	0.02
β-blocker initiation	0.81 (0.58- 1.12)	0.21
CCB initiation	0.78 (0.57- 1.08)	0.14
Thiazide diuretic initiation	0.96 (0.64- 1.44)	0.85
Other class initiation	0.78 (0.53- 1.15)	0.21
**Adjustment for variables with univariate association with outcome**		
ACEI or ARB initiation	1.65 (1.16- 2.34)	0.006
β-blocker initiation	0.87 (0.607- 1.24)	0.43
CCB initiation	0.89 (0.626- 1.26)	0.51
Thiazide diuretic initiation	1.00 (0.646- 1.55)	0.99
Other class initiation	0.87 (0.57- 1.33)	0.52
**Exclusion of subjects non-compliant with initiated medications at 90-day follow up**		
ACEI or ARB initiation	1.89 (1.17- 3.08)	0.01
β-blocker initiation	0.95 (0.593- 1.52)	0.83
CCB initiation	0.59 (0.369- 0.96)	0.03
Thiazide diuretic initiation	1.06 (0.534- 2.10)	0.87
Other class initiation	0.61 (0.34- 1.10)	0.10
**Specification of DHP-CCB**		
ACEI or ARB initiation	1.49 (1.08- 2.05)	0.02
β-blocker initiation	0.82 (0.59- 1.13)	0.21
DHP-CCB initiation	0.82 (0.60- 1.13)	0.22
Thiazide diuretic initiation	1.01 (0.68- 1.51)	0.96
Other class initiation	0.79 (0.54- 1.16)	0.22
**Separation of ACEI and ARB effect**		
ACEI initiation	1.37 (0.99- 1.90)	0.06
ARB initiation	2.66 (1.24- 5.69)	0.01
β-blocker initiation	0.84 (0.61- 1.16)	0.28
CCB initiation	0.84 (0.61- 1.15)	0.28
Thiazide diuretic initiation	1.03 (0.69- 1.55)	0.88
Other class initiation	0.77 (0.52- 1.13)	0.18
**Effect of all discharged antihypertensives (initiated and continued)**		
Discharge ACEI or ARB	1.84 (1.109- 3.04)	0.02
Discharge β-blocker	1.08 (0.641- 1.82)	0.77
Discharge CCB	1.14 (0.681- 1.91)	0.62
Discharge Thiazide diuretic	1.34 (0.782- 2.29)	0.29
Discharge Other class	1.01 (0.55- 1.87)	0.97
**Association with mRS 0-2 at discharge**		
ACEI or ARB initiation	0.86 (0.60- 1.24)	0.43
β-blocker initiation	0.79 (0.54- 1.14)	0.21
CCB initiation	0.67 (0.47- 0.96)	0.03
Thiazide diuretic initiation	1.02 (0.66- 1.58)	0.94
Other class initiation	0.49 (0.31- 0.79)	0.003
**Analysis using logistic regression model without random effect by site**		
ACEI or ARB initiation	1.50 (1.09- 2.07)	0.01
β-blocker initiation	0.82 (0.59- 1.13)	0.22
CCB initiation	0.80 (0.58- 1.10)	0.17
Thiazide diuretic initiation	1.01 (0.67- 1.51)	0.97
Other class initiation	0.78 (0.53- 1.15)	0.21

© 2025 Ridha M et al. *JAMA Network Open.*

Abbreviations: ACEI, angiotensin converting enzyme inhibitor; ARB, angiotensin II receptor block; CCB, calcium channel blocker; DHP, dihydropyridine.

[a] Results of fully adjusted multivariable models are shown.

© 2025 Ridha M et al. *JAMA Network Open.*

eTable 5. Association of Antihypertensive Class With Favorable Outcome in Lobar Intracerebral Hemorrhage

Characteristic	AOR (95% CI)	P-value
Age per y	0.97 (0.95- 0.98)	<0.001
Sex		
Female	0.66 (0.42- 1.05)	0.08
Male	1 [Reference]	NA
Race and ethnicity [a]		
Hispanic	0.46 (0.27- 0.78)	0.004
Black	0.67 (0.37- 1.21)	0.18
White	1 [Reference]	NA
Prior stroke	0.44 (0.23- 0.83)	0.01
Diabetes	0.83 (0.50- 1.38)	0.47
Coronary artery disease	0.85 (0.44- 1.63)	0.62
Atrial fibrillation	0.69 (0.32- 1.52)	0.36
Heart failure	0.88 (0.36- 2.17)	0.78
Creatinine (per mg/dL increase)	0.94 (0.80- 1.12)	0.51
Log-transformed hematoma volume, baseline	0.18 (0.11- 0.32)	<0.001
IVH presence	0.80 (0.47- 1.36)	0.41
mRS score prior to ICH	0.60 (0.46- 0.77)	<0.001
GCS score at admission	1.08 (1.00- 1.17)	0.05
MAP at enrollment per mm Hg increase	1.01 (0.99- 1.03)	0.24
Total No. of antihypertensive medications prescribed at discharge	0.91 (0.72- 1.15)	0.41
ACEI or ARB initiation	0.65 (0.37- 1.13)	0.12
β-blocker initiation	0.75 (0.43- 1.30)	0.30
CCB initiation	1.05 (0.60- 1.84)	0.86
Thiazide diuretic initiation	0.52 (0.24- 1.16)	0.11
Other initiation	1.26 (0.60- 2.64)	0.54

Abbreviations: ACEI, angiotensin converting enzyme inhibitor; ARB, angiotensin II receptor block; CCB, calcium channel blocker; GCS, Glasgow Coma Scale; ICH, intracerebral hemorrhage; IVH, intraventricular hemorrhage; MAP, mean arterial pressure.

[a] Reference is non-Hispanic white.

© 2025 Ridha M et al. *JAMA Network Open.*

eFigure 1. Flowchart of Cohort Derivation

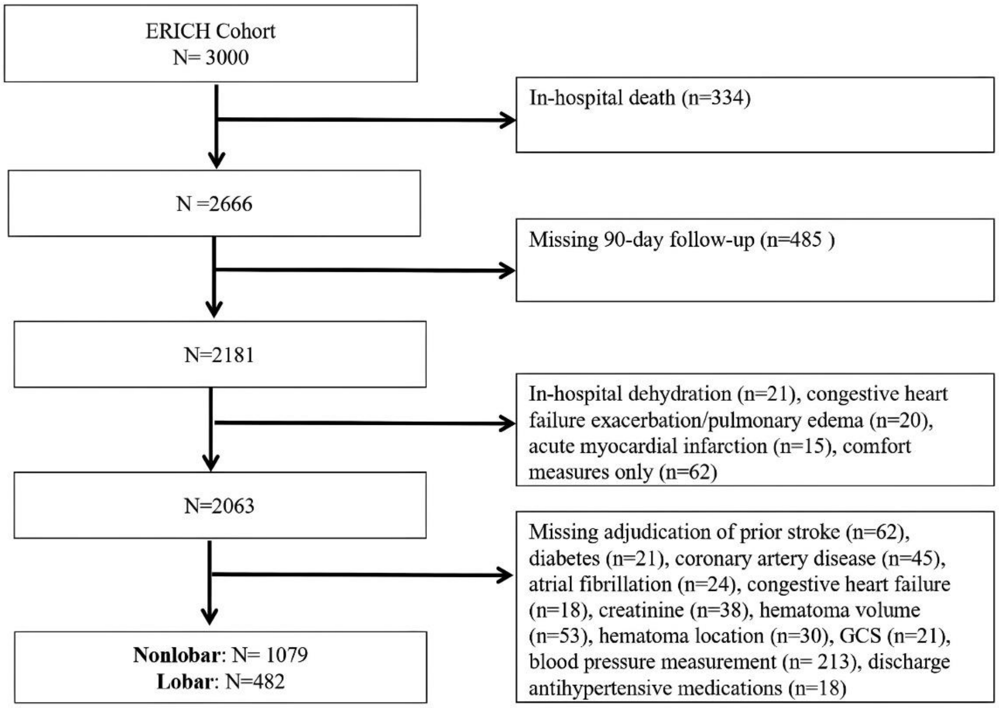

ERICH-Ethnic/Racial Variations of Intracerebral Hemorrhage Study

eFigure 1 Legend: Of the 3000 cases enrolled in ERICH, 334 died during hospitalization, 485 did not have available 90-day outcome, 56 experienced an in-hospital complications limiting choice of antihypertensive medication, 62 were transitioned to comfort-measures-only, and 543 were missing covariate data. Of the remaining 1561 subjects, 1079 had non-lobar and 482 had lobar intracerebral hemorrhage.

© 2025 Ridha M et al. *JAMA Network Open.*

eFigure 2. Association Between Renin-Angiotensin System Inhibitor Initiation and Distribution of 90-Day Modified Rankin Scale After Non-Lobar Intracerebral Hemorrhage

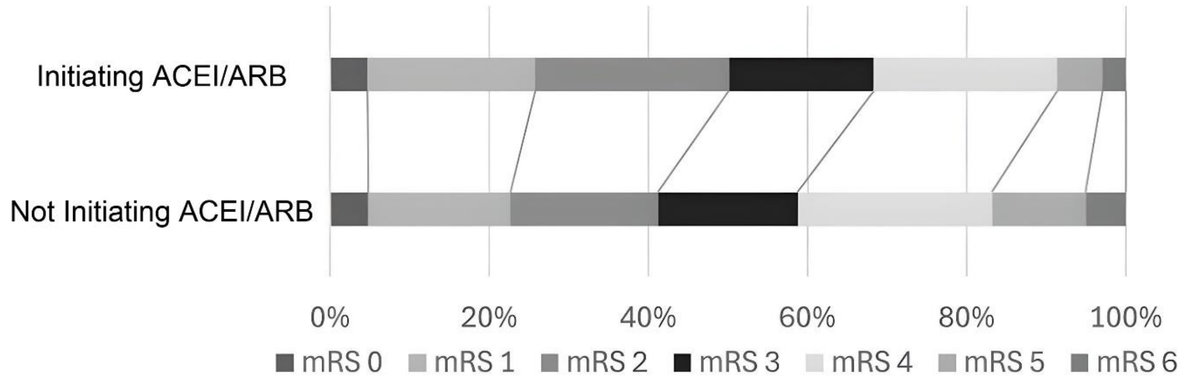

eFigure 2 legend: ACEI, angiotensin-converting enzyme inhibitor; ARB, angiotensin II receptor blocker; mRS, modified Rankin Scale.

© 2025 Ridha M et al. *JAMA Network Open.*
