# Supplementary material for: Antihypertensive Medication Class and Functional Outcomes After Nonlobar Intracerebral Hemorrhage
Source: JAMA Netw Open. 2025 Feb 3;8(2):e2457770. doi: 10.1001/jamanetworkopen.2024.57770 (PMC11791703; doi:10.1001/jamanetworkopen.2024.57770)
Supplement: Supplement 2. — Data Sharing Statement [file jamanetwopen-e2457770-s002.pdf]

Data Sharing Statement

Ridha. Antihypertensive Medication Class and Functional Outcomes After Nonlobar Intracerebral Hemorrhage. *JAMA Netw Open*. Published February 03, 2025. doi:10.1001/jamanetworkopen.2024.57770

Data
Data available: Yes
Data types: Deidentified participant data
How to access data: Data for this analysis is available upon request to the NINDS clinical archives dataset available at: https://www.ninds.nih.gov/current-research/research-funded-ninds/clinical-research/archived-clinical-research-datasets
When available: With publication

Supporting Documents
Document types: None

Additional Information
Who can access the data: Qualified researcher with approved proposal.
Types of analyses: Any purpose approved by review committee.
Mechanisms of data availability: After approval of proposal
